# Minimal Association Between Immunoglobulin A Coating and Gut Microbiota Alterations Induced by High-Fat Diets with Distinct Fatty Acid Compositions

**DOI:** 10.3390/ijms27062645

**Published:** 2026-03-13

**Authors:** Mao Teraoka, Naoki Nishino, Tianyang Wang, Kuiyi Chen, Takeshi Tsuruta

**Affiliations:** 1 Faculty of Environmental, Life, Natural Science and Technology, Okayama University, Okayama 7008530, Japan; mao.teraoka@s.okayama-u.ac.jp (M.T.); j1oufeed@okayama-u.ac.jp (N.N.); pvh11tk5@s.okayama-u.ac.jp (T.W.); pfiw96jr@s.okayama-u.ac.jp (K.C.); 2 Research Center for Intestinal Health Science, Okayama University, Okayama 7008530, Japan

**Keywords:** immunoglobulin A, high-fat diet, gut microbiota, fatty acid composition

## Abstract

High-fat diets (HFDs) containing dietary fats with different fatty acid (FA) compositions alter gut microbiota composition in a fat-source-dependent manner. Immunoglobulin A (IgA) and unabsorbed lipids in the distal gut are potential regulators of the gut microbiota. However, their roles in mediating gut microbiota alterations induced by dietary fats with different FA compositions remain unclear. This study aims to examine the associations of these two factors with fat-source-dependent gut microbiota alterations. BALB/c mice were fed a normal diet, a high-lard diet, a high-olive oil diet, or a high-soybean oil diet for 27 weeks. Fecal samples were collected to assess microbiota composition, the IgA coating index (ICI)—which quantifies the extent of IgA coating on gut microbiota—and fecal fatty acid concentrations. At the phylum level, the concentration of fecal total long-chain fatty acids (LCFAs) was positively correlated with the relative abundance (RA) of Bacillota and negatively correlated with that of Bacteroidota. In addition, a trend toward a positive association between the RA and the ICI was observed for Bacillota but not for Bacteroidota. At the genus level, the RAs of 12 taxa were positively correlated with fecal LCFA concentrations, whereas those of 6 taxa were negatively correlated. Although the RAs of most taxa appeared to be influenced by unabsorbed lipids and additional factors, only four Bacillota genera exhibited a positive correlation between the RA and the ICI. Our observations suggest that IgA coating of the gut microbiota may have a minimal association with fat-source-specific alterations in gut microbiota composition during HFD intake.

## 1. Introduction

There is a well-established association between habitual dietary patterns and gut microbiota composition [1]. Notably, dietary fat is one of the key components affecting the gut microbiota, and excessive intake has been shown to cause significant alterations in gut microbial composition—often termed dysbiosis—in both animal and human studies. In animal studies, a high-fat diet (HFD) supplemented with lard, an animal fat, is commonly used to assess the impact of excessive dietary fat intake on gut microbiota and host metabolism. High-lard diet intake has frequently been associated with an increase in the phylum Bacillota and a decrease in the phylum Bacteroidota, major bacterial phyla in the gut [2,3]. At the genus level, it has been shown to increase genera such as *Bilophila*, *Blautia*, *Enterorhabdus*, *Erysipelatoclostridium*, *Escherichia*–*Shigella*, *Harryflintia*, and *Lachnoclostridium*, while decreasing genera such as *Akkermansia*, *Bacteroides*, *Butyrivibrio*, Lachnospiraceae NK4A136 group, Muribaculaceae, and *Roseburia*, although some variations have been reported [2,3,4,5]. Excessive intake of dietary fats other than animal fat has also been reported to alter the gut microbiota composition in a fat-source-dependent manner [6,7,8]. An et al. reported that HFDs containing lard or 1 of 11 different plant-derived oils were administered to mice to compare their effects on the gut microbiota. They found that alpha diversity was significantly reduced in the high-lard diet group compared to the standard diet, whereas no marked decrease was observed in the groups fed high-olive oil or high-soybean oil diets. Furthermore, the ratio of the phylum Bacillota to Bacteroidota was highest in the high-lard diet group, whereas varying degrees of reduction were observed in the groups fed HFDs containing different plant oils [7].

HFD feeding induces profound shifts in the gut microbiota largely through bile acid-dependent mechanisms [9,10,11]. Increased dietary fat intake stimulates hepatic bile acid production and gut bile acid delivery, which in turn imposes strong selective pressure on the gut microbiota. Both primary and secondary bile acids display potent antimicrobial activities, directly inhibiting the growth of bile-sensitive taxa while promoting the expansion of bile-resistant microorganisms. As a result, bile acids are now regarded as a central mediator linking dietary fat intake to microbial community restructuring. In addition to bile acids, other luminal and host-derived factors, including unabsorbed dietary lipids and immunoglobulin A (IgA), have been suggested to contribute to HFD-induced dysbiosis. De Wit et al. reported that mice fed HFDs containing palm oil, olive oil, or soybean oil showed varying amounts of lipid reaching the lower gut and distinct differences in gut microbiota composition. Notably, the high-palm oil diet group exhibited the greatest lipid accumulation in the lower gut, along with reduced α-diversity and a marked decrease in Bacteroidetes [12]. Furthermore, free fatty acids (FAs), which are released from lipids by enzymatic action, exhibit potent antibacterial activity [13]. IgA is the principal antibody secreted into the gut lumen and is thought to modulate the gut microbiota composition via bacterial coating [14]. In patients with inflammatory bowel disease (IBD) and spondyloarthritis, increased IgA coating of pro-inflammatory bacteria has been reported in fecal samples compared to healthy individuals, suggesting that IgA binding to pathobionts may play a role in their regulation [15,16]. Conversely, in the case of certain commensal bacteria, IgA has been reported to support stable host–microbiota interactions by facilitating immune tolerance responses [17] and by modulating bacterial gene expression and metabolic functions associated with symbiosis [18]. Furthermore, we previously demonstrated that intake of a high-lard diet clearly alters the IgA coating profiles of the gut microbiota compared to a normal-fat diet in mice [19]. However, it remains unclear to what extent the levels of unabsorbed lipids in the distal gut and the IgA coating of the gut microbiota each associate with fat-source-dependent gut microbiota alterations. We aimed to examine the associations of these two factors by assessing fecal microbiota composition, IgA coating profiles of fecal microbiota, and fecal FA concentrations derived from diverse lipid species—namely, glycerolipids, phospholipids, cholesteryl esters, and free FAs—in mice fed HFDs formulated with dietary fats differing in FA composition and digestive properties.

## 2. Results

### 2.1. Fecal FA Concentrations

**Figure 1:**
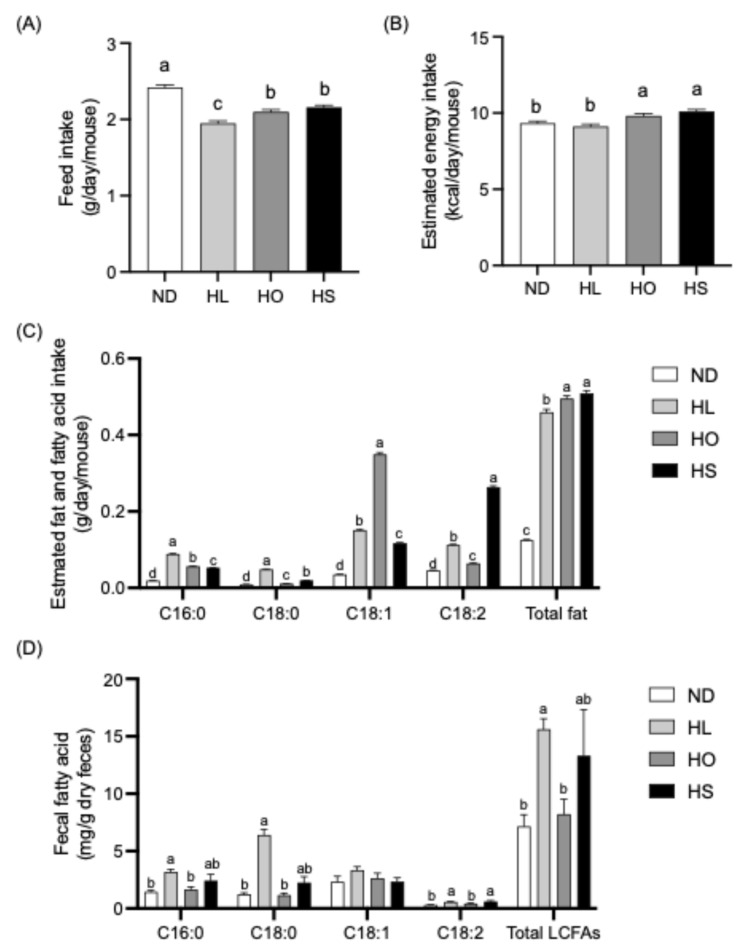
(**A**) Feed intake; (**B**) estimated energy intake; (**C**) estimated fat and fatty acid intake; (**D**) fecal fatty acid concentrations. One-way ANOVA or Kruskal–Wallis test followed by a post hoc multiple-comparisons test was conducted to compare differences among the ND, HL, HO, and HS groups for each parameter. Data are presented as the mean ± SEM (*n* = 6 per group). Values without a shared letter were significantly different (*p* < 0.05). Abbreviations: ND: normal diet; HL: high-lard diet; HO: high-olive oil diet; HS: high-soybean oil diet; LCFAs: long-chain fatty acid.

The feed intake, estimated energy intake, and estimated fat and FA intake are shown in Figure 1A–C, respectively. The feed intake was significantly lower in the HL, HO, and HS groups than in the ND group, with the lowest intake observed in the HL group (Figure 1A). The estimated energy intake was significantly lower in the ND and HL groups than in the HO and HS groups (Figure 1B). Estimated FA intake differed significantly among the ND, HL, HO, and HS groups for palmitic acid (C16:0), stearic acid (C18:0), oleic acid (C18:1), and linoleic acid (C18:2). For C16:0 and C18:0 intake, the HL group showed significantly higher values than the ND, HO, and HS groups. C18:1 intake was significantly elevated in the HO group relative to the ND, HL, and HS groups. In contrast, C18:2 intake was significantly higher in the HS group compared with the other groups, followed by the HL, HO, and ND groups. The estimated fat intake was significantly lower in the HL, HO, and HS groups than in the ND group, with the highest intake observed in the HO and HS groups (Figure 1C). The fecal concentrations of C16:0, C18:0, C18:1, C18:2, and long-chain fatty acids (LCFAs) are shown in Figure 1D. The HL group had significantly higher C16:0 and C18:0 concentrations than the ND and HO groups and tended to have a higher C18:0 concentration compared to the HS group (*p* = 0.06). The HL group also showed a trend toward a higher C18:1 concentration compared to the ND group (*p* = 0.08). The HL and HS groups exhibited significantly higher C18:2 concentrations than the ND and HO groups. The total LCFA concentration was significantly higher in the HL group than in the ND and HO groups.

### 2.2. Correlations Between RAs of Bacillota and Bacteroidota and Fecal FA Concentrations

**Figure 2:**
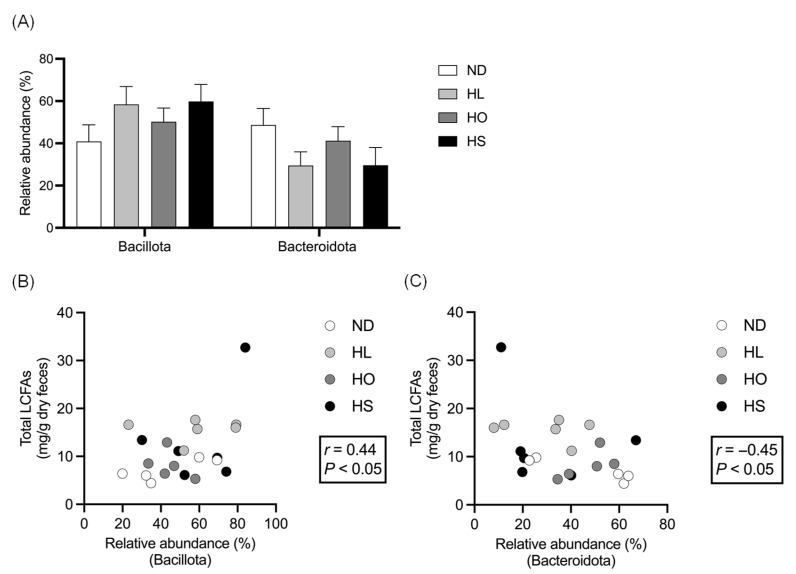
(**A**) Relative abundances of Bacillota and Bacteroidota in feces; correlations between fecal total long-chain fatty acids concentration and relative abundances of (**B**) Bacillota and (**C**) Bacteroidota. Data are presented as the mean ± SEM (*n* = 6 per group). Abbreviations: ND: normal diet; HL: high-lard diet; HO: high-olive oil diet; HS: high-soybean oil diet; LCFAs: long-chain fatty acids.

The RAs of Bacillota and Bacteroidota in feces are shown in Figure 2A. The RA of Bacillota tended to be higher in the HS group than in the ND group (*p* = 0.09). In contrast, the RA of Bacteroidota tended to be lower in the HL (*p* = 0.06) and HS (*p* = 0.07) groups compared with the ND group. The RA of Bacillota was significantly and positively correlated with the total LCFA concentration (*r* = 0.44, Figure 2B), whereas the RA of Bacteroidota was significantly and negatively correlated with the total LCFA concentration (*r* = −0.45, Figure 2C). No significant correlations were observed between the RAs of Bacillota or Bacteroidota and the fecal concentrations of individual FAs, including C16:0, C18:0, C18:1, and C18:2.

### 2.3. Correlations Between RAs and ICIs of Bacillota and Bacteroidota

**Figure 3:**
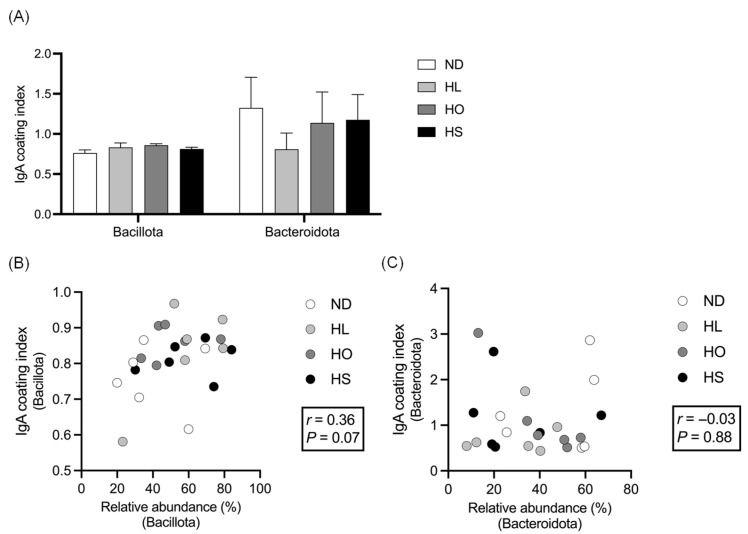
(**A**) IgA coating indices of Bacillota and Bacteroidota in feces; correlations between IgA coating indices and relative abundances of (**B**) Bacillota and (**C**) Bacteroidota. Data are presented as the mean ± SEM (*n* = 6 per group). Abbreviations: ND: normal diet; HL: high-lard diet; HO: high-olive oil diet; HS: high-soybean oil diet.

The ICIs of Bacillota and Bacteroidota in feces are shown in Figure 3A. The ICI of Bacillota tended to be higher in the HO group than in the ND group (*p* = 0.08). No significant difference in the ICI of Bacteroidota was observed among the groups. The ICI of Bacillota tended to be positively correlated with the RA of Bacillota (*r* = 0.36, *p* = 0.07, Figure 3B), whereas there was no significant correlation between the ICI and the RA of Bacteroidota (*r* = −0.03, *p* = 0.88, Figure 3C).

### 2.4. Correlations Between RAs of Fecal Bacteria at Genus Level and Fecal FA Concentrations

The correlations between the RAs of fecal bacteria at the genus level and the fecal FA concentrations are shown in Table 1. The fecal FA concentrations were significantly and positively correlated with the RA of *Acetatifactor* (C18:0), *Bilophila* (C18:0, C18:2), *Dorea* (C16:0, C18:0), Eubacterium coprostanoligenes group (C16:0, C18:0, C18:1), Eubacterium xylanophilum group (C18:2), *Harryflintia* (C16:0, C18:0), *Intestinimonas* (C16:0, C18:0, C18:1), Lachnospiraceae A2 (total LCFAs), Lachnospiraceae FCS020 group (C16:0), Unclassified (UC) Lachnospiraceae 1 (C16:0, C18:0, C18:1, total LCFAs)*,* UC Lachnospiraceae 2 (C16:0, total LCFAs), and UC Oscillospiraceae (C16:0, C18:0, C18:1). In contrast, negative correlations were observed between the fecal FA concentrations and the RA of *Akkermansia* (C16:0, C18:0), Clostridia UCG-014 (C18:0), Muribaculaceae (C16:0, total LCFAs), *Romboutsia* (C16:0, C18:0, C18:1, C18:2)*,* Ruminococcaceae UBA1819 (C18:0), and UC Erysipelotrichaceae 1 (C18:1).

### 2.5. Correlations Between RAs and ICIs of Fecal Bacteria at Genus Level

The ICIs of fecal bacteria at the genus level are shown in Table 2. The ND group exhibited a significantly higher ICI for *Anaerovorax* (compared to the HL group), *Blautia* (compared to the HS group), *Colidextribacter* (compared to the HL group), Defluviitaleaceae UCG-011 (compared to the HL and HS groups), Oscillospirales UCG-010 (compared to the HS group), and *Romboutsia* (compared to the HS group). The ND group exhibited a significantly lower ICI for *Alistipes* (compared to the HL and HO groups), *Anaerotruncus* (compared to the HO group), Clostridia vadin BB60 group (compared to the HO group), Clostridium sensu stricto 1 (compared to the HS group), *Erysipelatoclostridium* (compared to the HO group), *Lachnoclostridium* (compared to the HO group), Lachnospiraceae A2 (compared to the HL, HO, and HS groups), *Mucispirillum* (compared to the HS group), and *Streptococcus* (compared to the HO group).

Details of the correlation between the RAs and the ICIs of fecal bacteria at the genus level are shown in Table 3. Significantly positive correlations were observed between the RAs and the ICIs for the Acetivibrio ethanolgignens group, Clostridia vadin BB60 group, *Monoglobus*, and UC Oscillospirales.

### 2.6. RAs of Fecal Bacteria at Genus Level

The RAs in feces at the genus level are shown in Table 4. The results were categorized into four groups. Group 1: Bacterial genera showing a significant positive correlation between their RAs and the fecal FA concentrations; Group 2: bacterial genera showing a significant negative correlation between their RAs and the fecal FA concentrations; Group 3: bacterial genera showing a significant correlation between their RAs and ICIs; Group 4: bacterial genera whose RAs were significantly altered by HFD intake but did not show significant correlations between their RAs and the fecal FA concentrations or their ICIs.

In Group 1, the RAs were higher in the HL group than in the ND and HO groups, especially in *Bilophila*, Eubacterium coprostanoligenes group, and UC Lachnospiraceae 1. In Group 2, the RAs were lower in the HL group than in the ND and HO groups, especially in *Akkermansia*, Clostridia UCG-014, and *Romboutsia*. In Group 3, the RA of Acetivibrio ethanolgignens group was significantly higher in the HL group than in the ND group. The RA of the Clostridia vadin BB60 group was significantly higher in the HO group than in the HL and HS groups. *Monoglobus* showed significantly higher RA in the HO and HS groups than in the HL group. The RA of UC Oscillospirales was significantly higher in the HL group than in the HO and HS groups. None of these bacterial genera overlapped with those classified as Group 1 or Group 2. In Group 4, the ND group exhibited a significantly higher RA of *Alistipes* (compared to the HS group), *Enterococcus* (compared to the HL group), UC Erysipelotrichaceae 2 (compared to the HL group), and *Ruminiclostridium* (compared to the HS group). The ND group exhibited a significantly lower RA of *Bifidobacterium* (compared to the HS group), Butyricicoccaceae UCG-009 (compared to the HO group), *Enterorhabdus* (compared to the HO and HS groups), *Lachnoclostridium* (compared to the HO and HS groups), Lachnospiraceae UCG-004 (compared to the HS group), *Lactococcus* (compared to the HO and HS groups), RF39 (compared to the HO group), *Streptococcus* (compared to the HO and HS groups), and *Tuzzerella* (compared to the HS group).

### 2.7. Differences in Microbial Community Structure Among Groups

**Figure 4:**
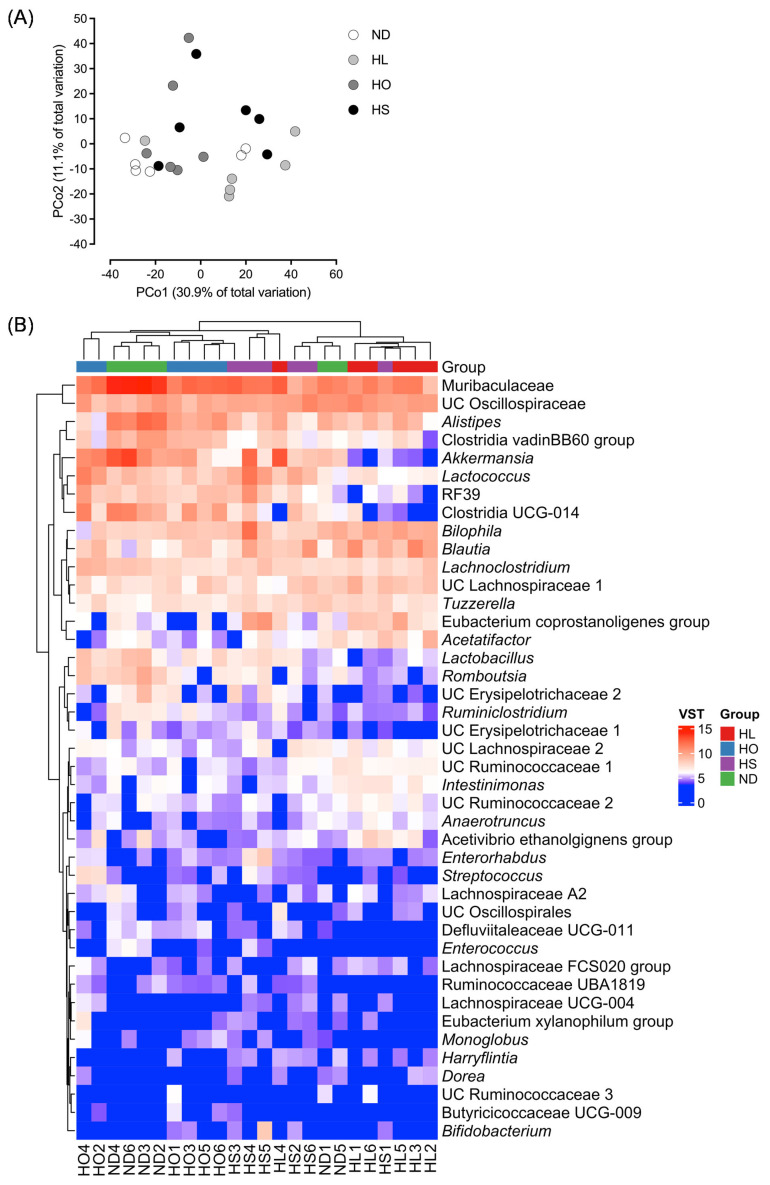
(**A**) Bray–Curtis β-diversity analysis at the genus level. Principal coordinates analysis (PCoA) based on Bray–Curtis dissimilarity calculated from genus-level relative abundance data using QIIME2. Each point represents an individual sample, and colors indicate experimental groups. Statistical significance of group separation was assessed using permutational multivariate analysis of variance. (**B**) Heatmap showing variance-stabilized genus-level abundance of selected genera across all individual samples. Both rows and columns were hierarchically clustered using Euclidean distance and complete linkage. Genera correspond to those listed in Table 4. Abbreviations: ND: normal diet; HL: high-lard diet; HO: high-olive oil diet; HS: high-soybean oil diet; VST: variance stabilizing transformation.

Bray–Curtis β-diversity analysis at the genus level is shown in Figure 4A. In this analysis, PERMANOVA revealed significant differences in the microbial community structure among the groups (pseudo-F = 2.23). Pairwise comparisons showed significant differences between the ND and HL groups and between the ND and HS groups, whereas a statistical tendency was observed between the ND and HO groups (*p* = 0.07). In contrast, the VST-normalized heatmap demonstrated variability in the abundance of taxa within each group. Although a certain degree of overlap in distribution among samples was observed, the HL and HS groups showed distinct abundance patterns compared with the ND group.

Hierarchical clustering analysis based on the RAs of the 42 genera listed in Table 4 is shown as a heatmap in Figure 4B. The heatmap, constructed using all individual samples, revealed that samples were broadly grouped according to dietary fat source, with HL and HS forming a distinct cluster from ND, despite some within-group variability. This clustering pattern was consistent with the separation observed in the β-diversity analysis.

## 3. Discussion

In this study, we examined the associations of unabsorbed lipids and IgA coating of the gut microbiota with fat-source-dependent gut microbiota alterations in mice fed HFDs formulated with dietary fats differing in FA composition and digestive properties. Although dietary fats are primarily absorbed in the small intestine, a fraction of dietary lipids can remain unabsorbed and reach the distal gut. Previous reviews have suggested that these unabsorbed lipid residues may contribute to shaping the colonic environment [20,21]. The fecal FA concentrations in each group did not necessarily directly reflect the FA composition of the dietary fats fed to each group, suggesting that differences in the digestive and absorptive properties of the respective dietary fats influenced the fecal FA profile. In the HL group, C18:0, which was present at a relatively low proportion in lard, exhibited the highest concentration in the feces. In contrast, although the HO group had a higher feed intake than the HL group and olive oil contained more than twice the amount of C18:1 compared with lard, no significant difference in the fecal C18:1 concentration was observed between the HO and HL groups. Mattson et al. demonstrated in rats that triglycerides (TGs) containing C18:0 may show lower apparent absorption than those containing C18:1, depending on FA positional distribution and dietary calcium levels [22]. Based on this evidence, C18:0 present in lard may be more susceptible to reduced absorption efficiency, thereby contributing to its preferential detection in feces. On the other hand, most of the C18:1 contained in the respective dietary fats may have been efficiently absorbed in the small intestine, which could account for the absence of detectable differences in fecal C18:1 concentrations among the groups. Although the estimated C18:2 intake was higher in the HS group than in the HL group, no significant difference was observed in fecal C18:2 levels between the groups. This finding may reflect the intrinsic property of C18:2, which is absorbed more efficiently in the gut than saturated LCFAs. Indeed, unsaturated LCFAs such as C18:2 have been reported to be more readily solubilized in bile salt micelles than saturated LCFAs [23]. In addition, C18:2 has been shown to be absorbed more efficiently in the proximal small intestine than C16:0 [24]. Therefore, the absence of increased fecal C18:2 excretion in the HS group despite the higher dietary intake of C18:2 may reflect the high micellar solubility of C18:2 and the resulting efficient gut absorption.

A significant positive correlation was observed between the fecal total LCFA concentration and the RA of Bacillota, while an inverse correlation was detected for Bacteroidota. In addition, Bacillota tended to show a positive association between the RA and the ICI, whereas no such relationship was evident in Bacteroidota. Collectively, these findings imply that HFD-associated changes in Bacillota composition may primarily be associated with unabsorbed lipids in the distal gut and weakly influenced by IgA coating, whereas Bacteroidota composition appears to be more closely associated with unabsorbed lipids than with IgA coating.

At the genus level, the RAs of 12 bacterial taxa (classified as Genera Group 1 in Table 4) were positively correlated with fecal LCFA concentrations. Pathogenic bacteria have evolved diverse mechanisms to evade the antimicrobial effects of exogenous LCFAs [25]. *Haemophilus influenzae* reduces FA uptake by inactivating FA transporter [26], whereas *Staphylococcus aureus* detoxifies or exports toxic FAs via modifying enzymes and efflux pumps [27,28]. Although it remains unknown whether these 12 taxa identified in this study possess LCFA evasion mechanisms, these genera may be well adapted to lipid-rich gut environments. Among the genera classified as Group 1, *Bilophilia* has been reported to exhibit tolerance to alterations in bile acid composition induced by HFD intake [29]. Devkota et al. showed that taurocholic acid, which is increased by the intake of an HFD based on animal fat (milk fat), selectively promotes the expansion of *Bilophilia wadsworthia*, suggesting a high degree of adaptation of this genus to bile acid- and lipid-rich gut environments. In our study, the RA of *Bilophilia* was significantly higher in the HL group than in the ND group, and similar patterns were observed among other taxa in Genera Group 1. Taken together, these findings suggest that bile acid adaptability contributes substantially to HFD-induced alterations in the RAs of the taxa in Genera Group 1.

In contrast, the RAs of six taxa (classified as Genera Group 2 in Table 4), including *Akkermansia*, Muribaculaceae, and *Romboutsia*, were negatively correlated with fecal LCFA concentrations. Consistent with previous reports [30,31,32], the RAs of *Akkermansia*, Muribaculaceae, and *Romboutsia* were decreased in the HL group compared with the ND and HO groups. Similarly, in the HS group, the RAs of these taxa also tended to be lower than those in the ND group. These observations raise the possibility that the antibacterial effects of FAs may be involved in the HFD-induced alterations in the RAs of the taxa in Genera Group 2. FAs are known to exert antimicrobial activity through multiple mechanisms, including direct interactions with bacterial membranes that disrupt membrane integrity, interfere with electron transport, and inhibit oxidative phosphorylation, ultimately suppressing bacterial growth or inducing cell death [33,34,35]. In addition to such direct antimicrobial effects, LCFAs may also exert indirect effects on specific gut microbiota by impairing mucin secretion. Among these, *Akkermansia muciniphila*, the major bacterial species of the genus *Akkermansia*, is a commensal gut microbe that utilizes mucin as its primary nutrient source. Gulhane et al. reported that saturated LCFAs associated with HFD induce endoplasmic reticulum stress in colonic goblet cells and are associated with reduced *Muc2* expression and impaired mucus barrier integrity. HFD feeding also correlated with a reduced abundance of *Akkermansia muciniphila*, suggesting that diet-induced mucus dysfunction may contribute to alterations in mucin-utilizing gut bacteria [36].

The ICI for Acetivibrio ethanolgignens group, Clostridia vadin BB60 group, *Monoglobus*, and UC Oscillospirales (classified as Genera Group 3 in Table 4)—all of which belong to the phylum Bacillota—showed positive correlations with their RA. IgA coating of commensal gut microbiota has been implicated in both immune exclusion [37] and the maintenance of mutualistic host–microbe relationships [17,18], depending on the bacterial taxon. Our data indicate that alterations in the IgA coating of selected genera within the phylum Bacillota, observed upon intake of HFDs containing dietary fats with different FA compositions, may support the establishment or maintenance of symbiotic interactions. Notably, no bacterial genera displayed a significant inverse association between the ICI and the RA. This observation suggests that the HFD-induced alterations in the IgA coating of gut microbiota may be unlikely to drive the exclusion of specific taxa. Although intake of HFDs containing dietary fats with different FA compositions significantly altered the ICI of 23 taxa, only 4 taxa showed a significant correlation between their RAs and the corresponding ICIs. In addition, none of the 18 taxa that were significantly correlated with fecal LCFA concentrations (Genera Groups 1 and 2 in Table 4) exhibited a significant association between their RAs and the corresponding ICIs. These findings suggest that alterations in the IgA coating profiles of the gut microbiota induced by HFDs containing dietary fats with different FA compositions have a weak association with fat-source-dependent gut microbiota alterations, whereas other factors, including the luminal levels of unabsorbed lipids in the distal gut, may also contribute to these differences.

While the RAs of 20 taxa (classified as Genera Group 4 in Table 4) were significantly altered by intake of HFDs containing dietary fats with different FA compositions, these RAs showed no significant correlations with the ICIs of the corresponding taxa or with the fecal LCFA concentrations. Among the 20 taxa, characteristic changes in the RAs were observed in the HO and HS groups compared with the HL and ND groups. Specifically, the HS group showed significant increases in *Bifidobacterium*, Lachnospiraceae UCG-004, and *Tuzzerella*, accompanied by significant decreases in *Alistipes* and *Ruminiclostridium*. In contrast, the HO group exhibited significant increases in *Lactobacillus*, Butyricicoccaceae UCG-009, and RF39. In addition, *Enterorhabdus*, *Lachnoclostridium*, *Lactococcus*, and *Streptococcus* were commonly increased in both the HO and HS groups. Although luminal bile acid composition was not assessed in the present study, previous reports have demonstrated that dietary fat sources can influence both the abundance and composition of bile acids. Therefore, the observed differences in microbial composition between the HO/HS and HL groups may be associated with bile acid-mediated effects [38]. In addition to bile acid-mediated effects, differences in FA composition may contribute to shifts in the colonic ecological niche, potentially influencing microbial community structure. Host epithelial metabolism has been shown to regulate mucosal oxygenation through mitochondrial respiration and FA β-oxidation [39], which may influence the growth of oxygen-sensitive obligate anaerobes. In this context, the increased abundance of aerotolerant or facultative taxa such as *Lactobacillus*, *Lactococcus*, and *Streptococcus* in the HO or HS groups, along with the reduction in obligate anaerobes including *Ruminiclostridium* and *Alistipes* in the HS group, may be consistent with alterations in the luminal redox environment. These host-driven niche changes may differentially affect microbial taxa and potentially explain the opposing trends observed in the HS group, such as increased *Bifidobacterium* despite reduced SCFA-producing taxa. However, the interpretation of changes observed in taxa such as *Tuzzerella* and RF39 remains limited, and further investigation is required to clarify their ecological relevance.

We acknowledge that our study has several limitations related to experimental design, mechanistic validation, and generalizability. First, the experimental diets differed in energy density due to the substitution of corn starch with dietary fat in the HFD groups. Under ad libitum feeding conditions, this resulted in significantly higher estimated energy intake and fat intake in the HO and HS groups compared with the ND and HL groups. Therefore, it cannot be completely excluded that differences in actual nutrient intake contributed to the observed alterations in gut microbiota composition. Future studies employing pair-feeding strategies may help to further clarify the independent effects of dietary fat quantity and FA composition. Second, the present study focused on fecal LCFAs as a representative fraction of unabsorbed lipids. Although lipid classes other than LCFAs, such as sterols and phospholipids, may also contribute to microbial alterations, their potential involvement in luminal microbial modulation remains to be elucidated. Third, fecal samples were used to assess both unabsorbed FAs and gut microbiota composition; however, they may not fully reflect the availability or chemical forms of lipids to which gut microbiota are exposed along the intestinal lumen. Fourth, the present study primarily describes associations between fecal LCFAs, IgA coating, and gut microbiota composition, and does not establish causal relationships between these factors. Experiments using *Aicda*-deficient mice (IgA-deficient mice) would allow direct comparison under conditions lacking IgA, thereby helping to examine IgA-dependent and IgA-independent effects and to clarify the underlying molecular mechanisms. Finally, this study was conducted in female mice using a limited number of dietary fat sources. As sex was not included as a biological variable, the conclusions drawn from this study should be interpreted within the context of the specific experimental conditions employed. Evaluation using a wider spectrum of HFDs, including an additional animal-derived fat with a distinct FA profile and a ω-3 polyunsaturated FA-rich oil, as well as studies incorporating both sexes, would further strengthen the generalizability of the findings.

## 4. Materials and Methods

### 4.1. Animal Experiment

Female BALB/c mice (5 weeks old) were obtained from Jackson Laboratory Japan (Kanagawa, Japan). Throughout this study, the animals were housed in a temperature-controlled environment under a 12 h light/dark cycle, with ad libitum access to food and water. After a 7-day acclimation period, mice were weighed and randomly assigned to one of four groups to ensure similar average body weights across groups. A total of 24 mice were used in this study. Each experimental group consisted of six mice housed in two cages (three mice per cage). The experimental groups were as follows: mice fed a normal diet (ND group); mice fed a high-lard diet (HL group); mice fed a high-olive oil diet (HO group); and mice fed a high-soybean oil diet (HS group). The ND (D12450J) and the HFDs (containing 45% kcal from fat) were purchased from Research Diets, Inc. (New Brunswick, NJ, USA). The actual compositions of the experimental diets and the FA profiles of each fat source are presented in Table 5 and Table 6, respectively. All experimental diets were formulated to maintain identical compositions across groups except for dietary fat and corn starch. In the HFD groups, dietary fat was increased by partially replacing corn starch to achieve differences in fat quantity and FA composition among the diets. During the 27-week intervention, mice were housed three per cage. Because the mice were maintained under ad libitum feeding conditions, daily feed intake was monitored throughout the experimental period. Estimated energy intake (kcal/day) was calculated by multiplying daily feed intake by the corresponding dietary energy density (kcal/g), which was determined based on Atwater conversion factors. Estimated dietary fat and FA intake was calculated by multiplying daily feed intake by the corresponding fat or FA content of each diet. At the end of the feeding period, a fresh fecal sample was collected from each mouse and stored at −20 °C until analysis. No predefined inclusion or exclusion criteria were applied during the study. All animals that completed the experimental period were included in the analyses. Throughout the 27-week intervention, no deaths or signs of severe health deterioration were observed in any experimental group.

### 4.2. Measurement of Fecal Fatty Acid Concentrations

Total lipids in the feces were extracted as described previously [19]. For the synthesis of fatty acid methyl esters (FAMEs), the dried lipids were mixed with 1 mL of 0.5 M methanol sodium hydroxide and heated under reflux at 85 °C for 10 min. After this mixture cooled to room temperature, 1 mL of 14% boron trifluoride-methanol solution (Tokyo Chemical Industry Co., Ltd. (TCI), Tokyo, Japan) was added, and the resulting mixture was heated under reflux at 85 °C for 5 min. After this mixture cooled to room temperature, 2 mL of hexane (TCI) and 7 mL of a saturated solution of sodium chloride (TCI) were added; following vigorous mixing, the resulting mixture was left on the bench for a few minutes to permit separation of the aqueous and organic layers. The hexane layer was recovered and subjected to gas chromatography (GC) on a system equipped with a flame ionization detector (FID; GC-2030; Shimadzu, Tokyo, Japan) and a capillary column (Supelco SP-2560, 100 m × 0.25 mm × 0.2 μm; Millipore/Sigma-Aldrich, St. Louis, MO, USA). The injector and detector temperatures were set at 250 °C. Helium was used as the carrier gas, at a flow rate of 1 mL min^−1^. Following injection (1 μL; split ratio, 100:1), the oven temperature was maintained at 180 °C. The FAMEs in each sample were identified based on the retention times of FAME standards (Supelco 37 Component FAME Mix; Millipore/Sigma-Aldrich). The content of individual fatty acids was calculated using the GC-FID chromatogram. Fecal FA concentrations were used as an indirect indicator of incomplete gut absorption of dietary lipids and potential lipid exposure in the distal gut.

### 4.3. 16S rRNA Gene Sequencing of IgA^+^ and IgA^−^ Bacteria in Feces

Each fecal pellet was suspended in 500 µL of phosphate-buffered saline (PBS) and subjected to centrifugation (100× *g*, 20 min, 4 °C) to clear the suspension. The resulting supernatant was transferred to a fresh tube and subjected to a second round of centrifugation (9000× *g*, 10 min, 4 °C) to pellet the bacteria. The resulting bacterial pellet was washed twice with PBS and used for the isolation of IgA-coated bacteria (IgA^+^ bacteria) and non-coated bacteria (IgA^−^ bacteria). IgA^+^ and IgA^−^ bacteria were isolated using an affinity purification method using magnetic beads, as previously reported [19]. DNA was extracted from IgA^+^ and IgA^−^ bacteria using the Nucleospin tissue XS kit (Macherey-Nagel, Duren, Germany) according to the manufacturer’s instructions. PCR amplification of bacterial 16S rRNA genes and purification of the PCR amplicons were performed as described previously [40]. The purified amplicons were subjected to paired-end sequencing (2 × 250 b) on an Illumina Miseq platform (Illumina, San Diego, CA, USA); sequencing was conducted at FASMAC Co., Ltd. (Kanagawa, Japan). The archived raw sequences were processed using Quantitative Insights into Microbial Ecology 2 (QIIME2, version 2021. 11) software. The raw sequences were filtered to remove reads at any site receiving a quality score < 25 and length < 135 bp. Sequences were joined by paired ends, and chimeric sequences were identified and removed using the dada2 package executed in the R software (version 4.1.2) [41,42]. High-quality and non-chimeric sequences were grouped into sequence variants. Taxonomy was assigned using the SILVA rRNA database, and bacterial clustering was analyzed at the phylum and genus levels [43]. Data were normalized using “qiime diversity alpha-rarefaction”. Relative abundances (RAs, expressed as percentages) at the phylum and genus levels were calculated (separately) for IgA^+^ and IgA^−^ bacteria. RAs (%) were calculated as the proportion of sequence reads assigned to each taxon relative to the total number of quality-filtered, non-chimeric reads per sample after dada2 denoising. To evaluate the degree of IgA coating of bacteria in each phylum and genus, the IgA coating index (ICI) was calculated as defined previously [44]. In short, the ICI was calculated by dividing the RA of IgA^+^ bacteria by the RA of IgA^−^ bacteria. When the RA of IgA^−^ bacteria was zero, the lowest value (0.001%) was substituted. When both of the RAs were zero, the ICI was judged as not detected, given that this ratio was not calculable.

### 4.4. 16S rRNA Gene Sequencing of Total Bacteria in Feces

Total bacterial DNA was extracted from the feces using a QIAamp Fast DNA Stool Mini Kit (QIAGEN, Tokyo, Japan) according to the manufacturer’s instructions. PCR amplification of 16S rRNA genes, purification of the PCR amplicons, sequencing using an Illumina MiSeq, and QIIME2 analysis were conducted using a workflow like that described in Section 2.3. RAs (%) in total bacteria were calculated at the phylum and genus levels.

For β-diversity analysis, Bray–Curtis dissimilarity matrices were calculated based on genus-level RA data using QIIME2. Principal coordinates analysis (PCoA) was performed to visualize differences in microbial community composition among the experimental groups. Statistical significance of group separation was evaluated using permutational multivariate analysis of variance (PERMANOVA) with 999 permutations.

Differential abundance analysis was performed using the DESeq2 package (version 1.50.2) in R (version 4.5.2). Raw count data at the genus level were used as input without rarefaction. Counts were normalized using the DESeq2 median-of-ratios method. Variance stabilizing transformation (VST) was applied to normalized counts to generate values suitable for visualization and clustering. Hierarchical clustering was performed based on Euclidean distance with complete linkage, and heatmaps were generated using the Complex Heatmap package. The genera listed in Table 4 were specifically selected for visualization in the heatmap to demonstrate their relative abundance patterns and hierarchical clustering among the experimental groups.

### 4.5. Statistical Analysis

All statistical analyses were conducted using Prism for Mac (version 10.00; GraphPad Software, San Diego, CA, USA). The results are presented as the mean ± SEM. The Shapiro–Wilk test was applied to test for normality. For data that failed the normality test, differences among groups were evaluated using the Kruskal–Wallis test. For data that passed the normality test, equality of variance was tested using Bartlett’s test. Based on the results of Bartlett’s test, differences among groups were evaluated using one-way analysis of variance (ANOVA) or the Kruskal–Wallis test. Where overall significance was returned, these tests were followed by post hoc multiple-comparisons tests adjusted by the false discovery rate using the method of Benjamini and Hochberg. Associations between each factor were evaluated using Pearson’s correlation for normally distributed data and Spearman’s rank correlation for non-normally distributed data. *p*-values < 0.05 were considered statistically significant, whereas results with 0.05 ≤ *p* < 0.10 were considered to indicate a statistical tendency. Exact *p*-values are reported where appropriate.

## 5. Conclusions

The present study is the first to indicate the associations of IgA coating of the gut microbiota with fat-source-specific alterations in gut microbiota composition during HFD intake. Our observations suggest that, under HFD conditions, IgA coating has a minimal association with fat-source-specific alterations in gut microbiota composition. In contrast, these alterations were more consistently associated with variations in unabsorbed lipid profiles in the distal gut.

Although the present findings were obtained under specific experimental conditions in female mice, they provide insight into how differences in dietary FA composition may influence gut microbial ecology through changes in the luminal lipid environment. Because dietary fat composition varies widely across human diets, including Western-style diets characterized by high intake of saturated and unsaturated FAs, the mechanisms suggested in this study may also contribute to microbiota variation across different dietary patterns. Furthermore, the observed associations between fecal LCFAs, IgA coating, and microbial composition highlight the potential importance of unabsorbed dietary lipids as modulators of host–microbiota interactions. Future studies examining these relationships across different host backgrounds, dietary contexts, and physiological conditions will be important to determine the extent to which these mechanisms operate beyond the specific experimental model used in this study and to clarify their potential relevance to human gut microbial ecology.

## Figures and Tables

**Table 1:** Correlations between relative abundances of fecal bacteria at genus level and fecal fatty acid concentrations.

		Fatty Acid				
Genus	Phylum	C16:0	C18:0	C18:1	C18:2	Total LCFAs
*Acetatifactor*	Bacillota	-	0.52	-	-	-
*Bilophila*	Pseudomonadota	-	0.44	-	0.49	-
*Dorea*	Bacillota	0.48	0.49	-	-	-
Eubacterium coprostanoligenes group	Bacillota	0.58	0.76	0.43	-	-
Eubacterium xylanophilum group	Bacillota	-	-	-	0.51	-
*Harryflintia*	Bacillota	0.48	0.44	-	-	-
*Intestinimonas*	Bacillota	0.45	0.45	0.44	-	-
Lachnospiraceae A2	Bacillota	-	-	-	-	0.44
Lachnospiraceae FCS020 group	Bacillota	0.46	-	-	-	-
UC Lachnospiraceae 1	Bacillota	0.54	0.48	0.45	-	0.56
UC Lachnospiraceae 2	Bacillota	0.45	-	-	-	0.44
UC Oscillospiraceae	Bacillota	0.57	0.57	0.44	-	-
*Akkermansia*	Verrucomicrobiota	−0.52	−0.61	-	-	-
Clostridia UCG-014	Bacillota	-	−0.58	-	-	-
Muribaculaceae	Bacteroidota	−0.43	-	-	-	−0.52
*Romboutsia*	Bacillota	−0.58	−0.53	−0.50	−0.51	-
Ruminococcaceae UBA1819	Bacillota	-	−0.47	-	-	-
UC Erysipelotrichaceae 1	Bacillota	-	-	−0.49	-	-

If the variables were normally distributed, Pearson’s correlation coefficient analysis was performed; otherwise, Spearman’s correlation coefficient analysis was used. Correlation coefficients are shown for the bacterial genera that exhibited significant correlations (*p* < 0.05). Abbreviations: LCFAs: Long chain fatty acids; UC: Unclassified.

**Table 2:** IgA coating index of fecal bacteria at genus level.

Genus	Phylum	ND	HL	HO	HS
*Alistipes*	Bacteroidota	1.34 ± 0.17 ^b^	2.54 ± 0.30 ^a^	3.86 ± 0.82 ^a^	4.76 ± 1.92 ^ab^
*Anaerotruncus*	Bacillota	0.06 ± 0.06 ^b^	0.15 ± 0.04 ^ab^	0.46 ± 0.19 ^a^	0.57 ± 0.47 ^ab^
*Anaerovorax*	Bacillota	0.40 ± 0.14 ^a^	0.03 ± 0.03 ^b^	0.24 ± 0.08 ^a^	0.16 ± 0.11 ^ab^
*Blautia*	Bacillota	4.36 ± 3.26 ^a^	1.20 ± 0.39 ^ab^	0.62 ± 0.13 ^ab^	0.51 ± 0.12 ^b^
Clostridia vadin BB60 group	Bacillota	1.55 ± 0.92 ^b^	0.54 ± 0.24 ^b^	3.81 ± 0.34 ^a^	1.80 ± 0.95 ^ab^
Clostridium sensu stricto 1	Bacillota	3.48 ± 0.34 ^b^	5.53 ± 0.83 ^ab^	4.69 ± 1.07 ^ab^	7.61 ± 1.48 ^a^
*Colidextribacter*	Bacillota	0.45 ± 0.04 ^a^	0.30 ± 0.05 ^b^	0.50 ± 0.10 ^ab^	0.33 ± 0.07 ^ab^
Defluviitaleaceae UCG-011	Bacillota	0.79 ± 0.13 ^a^	0.21 ± 0.15 ^b^	0.61 ± 0.10 ^ab^	0.24 ± 0.13 ^b^
*Enterorhabdus*	Actinobacteriota	0.47 ± 0.11 ^ab^	0.28 ± 0.07 ^b^	0.60 ± 0.12 ^a^	0.87 ± 0.43 ^ab^
*Erysipelatoclostridium*	Bacillota	5.73 ± 1.44 ^b^	3.61 ± 0.96 ^b^	15.81 ± 2.38 ^a^	4.65 ± 0.87 ^b^
*Lachnoclostridium*	Bacillota	0.85 ± 0.34 ^b^	1.25 ± 0.30 ^ab^	1.40 ± 0.30 ^a^	0.86 ± 0.21 ^ab^
Lachnospiraceae A2	Bacillota	0.27 ± 0.05 ^b^	1.08 ± 0.20 ^a^	1.00 ± 0.15 ^a^	5.45 ± 4.56 ^a^
Lachnospiraceae GCA-900066575	Bacillota	2.04 ± 1.34 ^ab^	0.53 ± 0.15 ^ab^	0.90 ± 0.14 ^a^	0.38 ± 0.11 ^b^
Lachnospiraceae NK4A136 group	Bacillota	1.21 ± 0.41 ^ab^	0.80 ± 0.10 ^b^	2.27 ± 0.65 ^a^	0.88 ± 0.19 ^b^
Lachnospiraceae UCG-006	Bacillota	0.49 ± 0.10 ^ab^	0.42 ± 0.11 ^ab^	0.73 ± 0.10 ^a^	0.26 ± 0.09 ^b^
*Monoglobus*	Bacillota	2.00 ± 1.04 ^ab^	0.04 ± 0.04 ^b^	2.37 ± 0.85 ^a^	0.64 ± 0.23 ^ab^
*Mucispirillum*	Deferribacterota	1.87 ± 1.00 ^b^	5.23 ± 1.66 ^ab^	4.59 ± 2.82 ^ab^	7.80 ± 2.43 ^a^
Oscillospirales UCG-010	Bacillota	3.80 ± 0.67 ^a^	2.63 ± 1.41 ^ab^	3.80 ± 0.90 ^a^	1.52 ± 0.63 ^b^
UC Oscillospiraceae	Bacillota	0.75 ± 0.11 ^ab^	0.53 ± 0.08 ^b^	0.85 ± 0.13 ^a^	0.61 ± 0.07 ^ab^
UC Oscillospirales	Bacillota	0.78 ± 0.61 ^ab^	1.32 ± 0.40 ^a^	0.54 ± 0.25 ^ab^	0.04 ± 0.04 ^b^
UC Peptococcaceae	Bacillota	0.78 ± 0.33 ^ab^	0.33 ± 0.11 ^b^	0.71 ± 0.06 ^a^	0.30 ± 0.07 ^b^
*Romboutsia*	Bacillota	2.27 ± 0.47 ^a^	1.88 ± 0.64 ^ab^	1.55 ± 0.47 ^ab^	0.54 ± 0.20 ^b^
*Streptococcus*	Bacillota	1.96 ± 1.28 ^b^	3.31 ± 1.75 ^ab^	7.38 ± 1.11 ^a^	2.10 ± 0.81 ^b^

Data are presented as the mean ± SEM (*n* = 6). One-way ANOVA or Kruskal–Wallis test followed by post hoc test was conducted to compare differences among the ND, HL, HO, and HS groups. Values without a shared letter were significantly different (*p* < 0.05). Bacterial genera that showed a significant difference among groups are shown. Abbreviations: ND: normal diet; HL: high-lard diet; HO: high-olive oil diet; HS: high-soybean oil diet; UC: unclassified.

**Table 3:** Correlations between relative abundances and IgA coating indices of fecal bacteria at genus level.

Genus	Phylum	*r* ^1^	*p* Value
Acetivibrio ethanolgignens group	Bacillota	0.69	<0.01
Clostridia vadin BB60 group	Bacillota	0.48	<0.05
*Monoglobus*	Bacillota	0.61	<0.05
UC Oscillospirales	Bacillota	0.62	<0.05

If the variables were normally distributed, Pearson’s correlation coefficient analysis was performed; otherwise, Spearman’s correlation coefficient analysis was used. Bacterial genera that showed a significant correlation (*p* < 0.05) are presented. ^1^ Correlation coefficient. Abbreviations: UC: Unclassified.

**Table 4:** Relative abundances of fecal bacteria at genus level.

	Genus	Phylum	ND	HL	HO	HS
Group 1	*Acetatifactor*	Bacillota	0.39 ± 0.22 ^ab^	1.72 ± 1.13 ^a^	0.08 ± 0.04 ^b^	0.79 ± 0.43 ^ab^
	*Bilophila*	Pseudomonadota	1.53 ± 0.75 ^b^	4.37 ± 1.23 ^a^	1.52 ± 0.31 ^b^	4.52 ± 2.05 ^ab^
	*Dorea*	Bacillota	0.01 ± 0.01	0.06 ± 0.02	0.01 ± 0.01	0.02 ± 0.01
	Eubacterium coprostanoligenes group	Bacillota	0.20 ± 0.08 ^bc^	2.00 ± 0.72 ^a^	0.14 ± 0.11 ^c^	2.34 ± 1.31 ^ab^
	Eubacterium xylanophilum group	Bacillota	0.01 ± 0.00	0.01 ± 0.01	0.10 ± 0.09	0.03 ± 0.01
	*Harryflintia*	Bacillota	0.06 ± 0.03 ^ab^	0.13 ± 0.03 ^a^	0.05 ± 0.02 ^b^	0.10 ± 0.03 ^ab^
	*Intestinimonas*	Bacillota	0.20 ± 0.10 ^ab^	0.48 ± 0.10 ^a^	0.17 ± 0.04 ^b^	0.31 ± 0.15 ^ab^
	Lachnospiraceae A2	Bacillota	0.08 ± 0.03	0.12 ± 0.04	0.09 ± 0.03	0.07 ± 0.04
	Lachnospiraceae FCS020 group	Bacillota	0.01 ± 0.01	0.08 ± 0.03	0.06 ± 0.03	0.07 ± 0.04
	UC Lachnospiraceae 1	Bacillota	5.80 ± 1.62 ^b^	13.29 ± 2.69 ^a^	6.40 ± 1.12 ^b^	8.24 ± 2.84 ^ab^
	UC Lachnospiraceae 2	Bacillota	0.94 ± 0.37	2.53 ± 0.76	1.34 ± 0.32	2.39 ± 0.72
	UC Oscillospiraceae	Bacillota	4.18 ± 1.65	8.09 ± 1.37	4.10 ± 0.64	7.79 ± 2.28
Group 2	*Akkermansia*	Verrucomicrobiota	6.77 ± 3.02 ^a^	4.72 ± 4.70 ^b^	5.82 ± 2.19 ^a^	3.68 ± 2.52 ^ab^
	Clostridia UCG-014	Bacillota	1.96 ± 0.73 ^a^	0.11 ± 0.11 ^b^	4.48 ± 1.56 ^a^	1.20 ± 0.56 ^ab^
	Muribaculaceae	Bacteroidota	30.87 ± 4.38 ^a^	15.70 ± 3.25 ^b^	24.63 ± 3.59 ^ab^	18.30 ± 5.17 ^b^
	*Romboutsia*	Bacillota	0.66 ± 0.15 ^a^	0.13 ± 0.03 ^b^	0.75 ± 0.26 ^a^	0.42 ± 0.13 ^ab^
	Ruminococcaceae UBA1819	Bacillota	0.01 ± 0.01 ^b^	0.003 ± 0.003 ^b^	0.04 ± 0.01 ^a^	0.02 ± 0.01 ^ab^
	UC Erysipelotrichaceae 1	Bacillota	0.12 ± 0.05 ^a^	0.01 ± 0.01 ^b^	0.04 ± 0.02 ^ab^	0.07 ± 0.04 ^ab^
Group 3	Acetivibrio ethanolgignens group	Bacillota	0.07 ± 0.04 ^b^	0.46 ± 0.19 ^a^	0.20 ± 0.15 ^ab^	0.32 ± 0.16 ^ab^
	Clostridia vadin BB60 group	Bacillota	1.52 ± 0.50 ^ab^	0.67 ± 0.21 ^b^	2.08 ± 0.47 ^a^	0.64 ± 0.23 ^b^
	*Monoglobus*	Bacillota	0.01 ± 0.01 ^ab^	0 ^b^	0.07 ± 0.03 ^a^	0.03 ± 0.01 ^a^
	UC Oscillospirales	Bacillota	0.02 ± 0.01 ^ab^	0.11 ± 0.06 ^a^	0.02 ± 0.01 ^b^	0.01 ± 0.003 ^b^
Group 4	*Alistipes*	Bacteroidota	5.59 ± 1.29 ^a^	2.71 ± 0.71 ^ab^	3.66 ± 1.22 ^ab^	1.46 ± 0.34 ^b^
	*Anaerotruncus*	Bacillota	0.14 ± 0.05 ^ab^	0.31 ± 0.09 ^a^	0.09 ± 0.04 ^b^	0.17 ± 0.06 ^ab^
	*Bifidobacterium*	Actinomycetota	0 ^b^	0 ^b^	0.02 ± 0.01 ^ab^	0.32 ± 0.28 ^a^
	*Blautia*	Bacillota	0.70 ± 0.49 ^b^	5.42 ± 1.75 ^a^	1.45 ± 0.43 ^ab^	4.20 ± 1.55 ^a^
	Butyricicoccaceae UCG-009	Bacillota	0 ^b^	0 ^b^	0.05 ± 0.03 ^a^	0.01 ± 0.01 ^ab^
	Defluviitaleaceae UCG-011	Bacillota	0.03 ± 0.01 ^ab^	0.02 ± 0.02 ^b^	0.06 ± 0.02 ^a^	0.03 ± 0.02 ^ab^
	*Enterococcus*	Bacillota	0.03 ± 0.01 ^a^	0 ^b^	0.003 ± 0.003 ^ab^	0.02 ± 0.01 ^ab^
	*Enterorhabdus*	Actinomycetota	0.01 ± 0.01 ^b^	0.04 ± 0.01 ^ab^	0.11 ± 0.03 ^a^	0.40 ± 0.28 ^a^
	UC Erysipelotrichaceae 2	Bacillota	0.34 ± 0.12 ^a^	0.08 ± 0.05 ^b^	0.22 ± 0.11 ^ab^	0.52 ± 0.21 ^a^
	*Lachnoclostridium*	Bacillota	0.71 ± 0.09 ^b^	1.54 ± 0.62 ^ab^	1.69 ± 0.53 ^a^	1.50 ± 0.24 ^a^
	Lachnospiraceae UCG-004	Bacillota	0.01 ± 0.01 ^b^	0.01 ± 0.01 ^b^	0.06 ± 0.03 ^ab^	0.06 ± 0.02 ^a^
	*Lactobacillus*	Bacillota	0.39 ± 0.11 ^ab^	0.18 ± 0.06 ^b^	0.84 ± 0.25 ^a^	0.47 ± 0.16 ^ab^
	*Lactococcus*	Bacillota	0.60 ± 0.11 ^c^	0.70 ± 0.13 ^bc^	4.27 ± 1.86 ^ab^	5.98 ± 1.94 ^a^
	RF39	Bacillota	0.49 ± 0.10 ^bc^	0.20 ± 0.09 ^c^	2.30 ± 0.63 ^a^	2.16 ± 0.86 ^ab^
	*Ruminiclostridium*	Bacillota	0.16 ± 0.04 ^a^	0.11 ± 0.04 ^ab^	0.17 ± 0.08 ^ab^	0.05 ± 0.01 ^b^
	UC Ruminococcaceae 1	Bacillota	0.69 ± 0.28 ^ab^	1.22 ± 0.25 ^a^	0.49 ± 0.15 ^b^	0.90 ± 0.26 ^ab^
	UC Ruminococcaceae 2	Bacillota	0.16 ± 0.06 ^ab^	0.28 ± 0.06 ^a^	0.11 ± 0.05 ^b^	0.15 ± 0.06 ^ab^
	UC Ruminococcaceae 3	Bacillota	0.03 ± 0.02 ^ab^	0 ^b^	0.08 ± 0.04 ^a^	0.03 ± 0.02 ^ab^
	*Streptococcus*	Bacillota	0.01 ± 0.004 ^b^	0.02 ± 0.01 ^ab^	0.38 ± 0.21 ^a^	0.08 ± 0.04 ^a^
	*Tuzzerella*	Bacillota	0.60 ± 0.27 ^b^	0.93 ± 0.12 ^ab^	0.67 ± 0.15 ^b^	1.28 ± 0.23 ^a^

Data are presented as the mean ± SEM (*n* = 6). One-way ANOVA or Kruskal–Wallis test followed by post hoc test was conducted to compare differences among the ND, HL, HO, and HS groups. Values without a shared letter were significantly different *p* < 0.05). Group 1: Bacterial genera showing a significant positive correlation between their RAs and the fecal FA concentrations. Group 2: Bacterial genera showing a significant negative correlation between their RAs and the fecal FA concentrations. Group 3: Bacterial genera showing a significant correlation between their RAs and ICIs. Group 4: Bacterial genera whose RAs were significantly altered by HFD intake but did not show significant correlations between their RAs and the fecal FA concentrations or their ICIs. Abbreviations: FA: fatty acid; ICI: IgA coating index; ND: normal diet; HL: high-lard diet; HO: high-olive oil diet; HS: high-soybean oil diet; RA: relative abundance; UC: unclassified.

**Table 5:** Composition of experimental diet.

Ingredient (g)	ND	HL	HO	HS
Casein	200	200	200	200
L-cystine	3	3	3	3
Soybean oil	25	0	0	202.5
Olive oil	0	0	202.5	0
Lard	30	202.5	0	0
Corn starch	506.2	151.8	151.8	151.8
Maltodextrin	125	125	125	125
Sucrose	68.8	68.8	68.8	68.8
Cellulose	50	50	50	50
Mineral mix	10	10	10	10
Dicalcium phosphate	13	13	13	13
Calcium carbonate	5.5	5.5	5.5	5.5
Potassium citrate, 1H_2_O	16.5	16.5	16.5	16.5
Vitamin mix	10	10	10	10
Choline bitrate	2	2	2	2
Energy density (kcal/g diet)	3.86	4.68	4.68	4.68

Abbreviations: ND: normal diet; HL: high-lard diet; HO: high-olive oil diet; HS: high-soybean oil diet.

**Table 6:** Fatty acid composition in dietary fats.

Fatty Acid (%)	Lard	Olive Oil	Soybean Oil
C16:0	19.3	11.5	10.4
C18:0	10.6	2.3	3.9
C18:1 *cis*	32.9	70.5	23.0
C18:2 *cis*	24.4	13.0	51.8
C18:3 *cis*	1.3	0.6	7.4
Others	11.5	2.1	3.6

## Data Availability

The original contributions presented in this study are included in the article. Further inquiries can be directed to the corresponding author.

## Abbreviations

The following abbreviations are used in this manuscript:

C16:0	Palmitic acid
C18:0	Stearic acid
C18:1	Oleic acid
C18:2	Linoleic acid
FA	Fatty acid
FAMEs	Fatty acid methyl esters
GC	Gas chromatography
HFD	High-fat diet
HL	High-lard diet
HO	High-olive oil diet
HS	High-soybean oil diet
IBD	Inflammatory bowel disease
ICI	IgA coating index
IgA	Immunoglobulin A
IgA^+^	IgA coated
IgA^−^	non-coated
LCFAs	Long-chain fatty acids
ND	Normal diet
PBS	Phosphate-buffered saline
RA	Relative abundance
TGs	Triglycerides
UC	Unclassified

## References

[1] Wu G.D., Chen J., Hoffmann C., Bittinger K., Chen Y.-Y., Keilbaugh S.A., Bewtra M., Knights D., Walters W.A., Knight R. (2011). Linking long-term dietary patterns with gut microbial enterotypes. Science.

[2] Mu H., Zhou Q., Yang R., Zeng J., Li X., Zhang R., Tang W., Li H., Wang S., Shen T. (2020). Naringin Attenuates High Fat Diet Induced Non-alcoholic Fatty Liver Disease and Gut Bacterial Dysbiosis in Mice. Front. Microbiol..

[3] Jo J.-K., Seo S.-H., Park S.-E., Kim H.-W., Kim E.-J., Kim J.-S., Pyo J.-Y., Cho K.-M., Kwon S.-J., Park D.-H. (2021). Gut microbiome and metabolome profiles associated with high-fat diet in mice. Metabolites.

[4] Schulz M.D., Atay Ç., Heringer J., Romrig F.K., Schwitalla S., Aydin B., Ziegler P.K., Varga J., Reindl W., Pommerenke C. (2014). High-fat-diet-mediated dysbiosis promotes intestinal carcinogenesis independently of obesity. Nature.

[5] Everard A., Belzer C., Geurts L., Ouwerkerk J.P., Druart C., Bindels L.B., Guiot Y., Derrien M., Muccioli G.G., Delzenne N.M. (2013). Cross-talk between *Akkermansia muciniphila* and intestinal epithelium controls diet-induced obesity. Proc. Natl. Acad. Sci. USA.

[6] Liu H., Zhu H., Xia H., Yang X., Yang L., Wang S., Wen J., Sun G. (2021). Different effects of high-fat diets rich in different oils on lipids metabolism, oxidative stress and gut microbiota. Food Res. Int..

[7] An J., Wang Q., Yi S., Liu X., Jin H., Xu J., Wen G., Zhu J., Tuo B. (2022). The source of the fat significantly affects the results of high-fat diet intervention. Sci. Rep..

[8] Abulizi N., Quin C., Brown K., Chan Y.K., Gill S.K., Gibson D.L. (2019). Gut Mucosal Proteins and Bacteriome Are Shaped by the Saturation Index of Dietary Lipids. Nutrients.

[9] Ridlon J.M., Kang D.J., Hylemon P.B., Bajaj J.S. (2014). Bile acids and the gut microbiome. Curr. Opin. Gastroenterol..

[10] Yokota A., Fukiya S., Islam K.B.M.S., Ooka T., Ogura Y., Hayashi T., Hagio M., Ishizuka S. (2012). Is bile acid a determinant of the gut microbiota on a high-fat diet?. Gut Microbes.

[11] David L.A., Maurice C.F., Carmody R.N., Gootenberg D.B., Button J.E., Wolfe B.E., Ling A.V., Devlin A.S., Varma Y., Fischbach M.A. (2014). Diet rapidly and reproducibly alters the human gut microbiome. Nature.

[12] De Wit N., Derrien M., Bosch-Vermeulen H., Oosterink E., Keshtkar S., Duval C., de Vogel-van den Bosch J., Kleerebezem M., Müller M., van der Meer R. (2012). Saturated fat stimulates obesity and hepatic steatosis and affects gut microbiota composition by an enhanced overflow of dietary fat to the distal intestine. Am. J. Physiol.-Gastrointest. Liver Physiol..

[13] Desbois A.P., Smith V.J. (2010). Antibacterial free fatty acids: Activities, mechanisms of action and biotechnological potential. Appl. Microbiol. Biotechnol..

[14] Kawamoto S., Tran T.H., Maruya M., Suzuki K., Doi Y., Tsutsui Y., Kato L.M., Fagarasan S. (2012). The inhibitory receptor PD-1 regulates IgA selection and bacterial composition in the gut. Science.

[15] Palm N.W., de Zoete M.R., Cullen T.W., Barry N.A., Stefanowski J., Hao L., Degnan P.H., Hu J., Peter I., Zhang W. (2014). Immunoglobulin A coating identifies colitogenic bacteria in inflammatory bowel disease. Cell.

[16] Viladomiu M., Kivolowitz C., Abdulhamid A., Dogan B., Victorio D., Castellanos J.G., Woo V., Teng F., Tran N.L., Sczesnak A. (2017). IgA-coated *E. coli* enriched in Crohn’s disease spondyloarthritis promote TH17-dependent inflammation. Sci. Transl. Med..

[17] Mikulic J., Longet S., Favre L., Benyacoub J., Corthesy B. (2017). Secretory IgA in complex with *Lactobacillus rhamnosus* potentiates mucosal dendritic cell-mediated Treg cell differentiation via TLR regulatory proteins, RALDH2 and secretion of IL-10 and TGF-β. Cell. Mol. Immunol..

[18] Nakajima A., Vogelzang A., Maruya M., Miyajima M., Murata M., Son A., Kuwahara T., Tsuruyama T., Yamada S., Matsuura M. (2018). IgA regulates the composition and metabolic function of gut microbiota by promoting symbiosis between bacteria. J. Exp. Med..

[19] Tsuruta T., Sonoyama K., Miyamoto T., Nguyen Q.D., Mizote A., Teraoka M., Nishino N. (2023). Cyclic Nigerosylnigerose Attenuates High-Fat Diet-Induced Fat Deposition, Colonic Inflammation, and Abnormal Glucose Metabolism and Modifies Gut Immunoglobulin a Reactivity to Commensal Bacteria. Mol. Nutr. Food Res..

[20] Michalski M.-C., Le Barz M., Vors C. (2021). Metabolic impact of dietary lipids: Towards a role of unabsorbed lipid residues?. OCL.

[21] Schoeler M., Caesar R. (2019). Dietary lipids, gut microbiota and lipid metabolism. Rev. Endocr. Metab. Disord..

[22] Mattson F.H., Nolen G.A., Webb M.R. (1979). The absorbability by rats of various triglycerides of stearic and oleic acid and the effect of dietary calcium and magnesium. J. Nutr..

[23] Smith A., Lough A.K. (1976). Micellar solubilization of fatty acids in aqueous media containing bile salts and phospholipids. Br. J. Nutr..

[24] Ockner R.K., Pittman J.P., Yager J.L. (1972). Differences in the intestinal absorption of saturated and unsaturated long chain fatty acids. Gastroenterology.

[25] Tchoupa A.K., Eijkelkamp B.A., Peschel A. (2022). Bacterial adaptation strategies to host-derived fatty acids. Trends Microbiol..

[26] Moleres J., Fernández-Calvet A., Ehrlich R.L., Martí S., Pérez-Regidor L., Euba B., Rodríguez-Arce I., Balashov S., Cuevas E., Liñares J. (2018). Antagonistic pleiotropy in the bifunctional surface protein FadL (OmpP1) during adaptation of *Haemophilus influenzae* to chronic lung infection associated with chronic obstructive pulmonary disease. mBio.

[27] Mortensen J., Shryock T., Kapral F. (1992). Modification of bactericidal fatty acids by an enzyme of *Staphylococcus aureus*. J. Med. Microbiol..

[28] Truong-Bolduc Q.C., Villet R.A., Estabrooks Z.A., Hooper D.C. (2014). Native Efflux Pumps Contribute Resistance to Antimicrobials of Skin and the Ability of *Staphylococcus aureus* to Colonize Skin. J. Infect. Dis..

[29] Devkota S., Wang Y., Musch M.W., Leone V., Fehlner-Peach H., Nadimpalli A., Antonopoulos D.A., Jabri B., Chang E.B. (2012). Dietary-fat-induced taurocholic acid promotes pathobiont expansion and colitis in *Il10^−/−^* mice. Nature.

[30] Koontanatechanon A., Wongphatcharachai M., Nonthabenjawan N., Jariyahatthakij P., Leksrisompong P., Srichana P., Prasopdee S., Roytrakul S., Sriyakul K., Thitapakorn V. (2022). The Effects of Increasing Dietary Fat on Serum Lipid Profile and Modification of Gut Microbiome in C57BL/6N Mice. J. Oleo Sci..

[31] Noureldein M.H., Rumora A.E., Teener S.J., Rigan D.M., Hayes J.M., Mendelson F.E., Carter A.D., Rubin W.G., Savelieff M.G., Feldman E.L. (2025). Dietary Fatty Acid Composition Alters Gut Microbiome in Mice with Obesity-Induced Peripheral Neuropathy. Nutrients.

[32] Schneeberger M., Everard A., Gómez-Valadés A.G., Matamoros S., Ramírez S., Delzenne N.M., Gomis R., Claret M., Cani P.D. (2015). *Akkermansia muciniphila* inversely correlates with the onset of inflammation, altered adipose tissue metabolism and metabolic disorders during obesity in mice. Sci. Rep..

[33] Galbraith H., Miller T. (1973). Effect of long chain fatty acids on bacterial respiration and amino acid uptake. J. Appl. Microbiol..

[34] Sheu C.W., Freese E. (1972). Effects of Fatty Acids on Growth and Envelope Proteins of *Bacillus subtilis*. J. Bacteriol..

[35] Wojtczak L., Wieckowski M.R. (1999). The Mechanisms of Fatty Acid-Induced Proton Permeability of the Inner Mitochondrial Membrane. J. Bioenerg. Biomembr..

[36] Gulhane M., Murray L., Lourie R., Tong H., Sheng Y.H., Wang R., Kang A., Schreiber V., Wong K.Y., Magor G. (2016). High Fat Diets Induce Colonic Epithelial Cell Stress and Inflammation that is Reversed by IL-22. Sci. Rep..

[37] Pabst O., Slack E. (2020). IgA and the intestinal microbiota: The importance of being specific. Mucosal Immunol..

[38] Sato Y., Furihata C., Matsushima T. (1987). Effects of high fat diet on fecal contents of bile acids in rats. Jpn. J. Cancer Res. GANN.

[39] Litvak Y., Byndloss M.X., Bäumler A.J. (2018). Colonocyte metabolism shapes the gut microbiota. Science.

[40] Muhomah T.A., Nishino N., Katsumata E., Haoming W., Tsuruta T. (2019). High-fat diet reduces the level of secretory immunoglobulin A coating of commensal gut microbiota. Biosci. Microbiota Food Health.

[41] Callahan B.J., McMurdie P.J., Rosen M.J., Han A.W., Johnson A.J., Holmes S.P. (2016). DADA2: High-resolution sample inference from Illumina amplicon data. Nat. Methods.

[42] The R Core Team (2021). A Language and Environment for Statistical Computing.

[43] Quast C., Pruesse E., Yilmaz P., Gerken J., Schweer T., Yarza P., Peplies J., Glöckner F.O. (2013). The SILVA ribosomal RNA gene database project: Improved data processing and web-based tools. Nucleic Acids Res..

[44] Takeuchi T., Miyauchi E., Kanaya T., Kato T., Nakanishi Y., Watanabe T., Kitami T., Taida T., Sasaki T., Negishi H. (2021). Acetate differentially regulates IgA reactivity to commensal bacteria. Nature.

